# The use and costs of health and social services in patients with longstanding substance abuse

**DOI:** 10.1186/1472-6963-13-185

**Published:** 2013-05-22

**Authors:** Corinna Vossius, Ingelin Testad, Rune Skjæveland, Sverre Nesvåg

**Affiliations:** 1 Stokka Teaching Nursing Home, Lassaveien 10, Stavanger, N-4022 Norway; 2 Centre for Age-related Medicine, Stavanger University Hospital, Stavanger, Norway; 3 Centre for Alcohol and Drug Research, Stavanger University Hospital, Stavanger, Norway

**Keywords:** Substance abuse, Costs, Health care services, Social services

## Abstract

**Background:**

Persons with longstanding substance abuse might become increasingly dependent on help by the public, eventually requiring permanent care. In 2006 the municipality of Stavanger established a so-called addiction ward for these clients, comprising 17 beds at the largest municipal nursing home. We assumed that the residents of this ward were high consumers of health care and social services during the last months preceding their admission. The aim of the study was to register the type and extent of services that were claimed by this client group during the last six months prior to admission, and to calculate the costs that were caused. Further, we estimated the incremental costs for nursing home placement.

**Methods:**

In 15 residents from the addiction ward the use of all welfare services during the six months prior to admission were registered. Costs were calculated by unit costs from a municipal, national and societal perspective.

**Results:**

Mean total costs during this period were €32 474. Approximately half of these costs were borne by state-funded institutions, and half were borne by the municipality. The clients used a great variety of services aimed at subsistence, health care and support in independent living, while services aimed at drug withdrawal were not claimed. There was no correlation between costs and the level of functioning. The incremental costs for nursing home admission were borne by the municipalities.

**Conclusion:**

Persons with longstanding substance abuse represent a group with a high use of welfare resources and hence cause high costs. However, our findings do not indicate any correlation between the amount of services rendered and the level of functioning. Further research should focus on the identification of the clients’ need for support in order to facilitate targeted interventions that might prevent further deterioration and, finally, the need for permanent care.

## Background

As compared to other European countries Norway has a low consume of alcohol and drugs [1]. Though it is difficult to give exact figures, the Norwegian Institute for Alcohol and Drug Research estimates that there are between 80 000 and 120 000 persons using high amounts of alcohol (2–3% of the population 16 years and older) and 10 000 to 18 000 (0.25–0.46% of the population 16 years and older) using heroin in Norway [2]. Ten of 100 000 inhabitants died of alcohol related diseases in 2011(1.2% of all deaths) [3].

Substance abuse is a challenge for every health care system because of the deteriorating effect of drug and alcohol not only on the health status [4,5], but on the ability to maintain common social skills and housing abilities, as well. Thus persons with longstanding substance abuse might become increasingly dependent on help by the public. When basic personal needs like nutrition and personal hygiene no longer can be met with the support by home care, these persons are normally placed in nursing homes, as these are the only institutions offering permanent care.

In the municipality of Stavanger with approximately 125 000 inhabitants this client group is gathered in a so-called addiction ward, where the use of legal substances is unprohibited, and where the treatment aim is not substance withdrawal but a strengthening of the residents’ remaining resources and a good quality of life. It was established in 2006 at the largest municipal nursing home next to six regular nursing home wards and comprises today 17 beds. During the last five years the addiction ward has become an established part of the health care sector in the municipality of Stavanger, as it provides a well-functioning care option for clients who formerly had been difficult to place [6].

However, public health care resources are scarce, and there is an ongoing discussion of whom to prioritize. Placement in a permanent care institution is one of the most expensive measures within a health care system and should be an effective step, either by meeting needs that cannot be satisfied in other ways or by replacing measures that are equally expensive. Therefore, the need for care is thoroughly evaluated in every patient, and clients have normally received various offers before, including home care and municipal housing supported by social workers. However, health care and social welfare are complex systems, and the financial sources might be municipal, governmental or private funding. There is no overstretching registration of services available, services rendered or costs involved, and there is little knowledge about the resources demanded by persons with longstanding substance abuse.

We assumed that the residents of the addiction ward were high consumers of health care and social services prior to their admission to the nursing home. The aim of the study was to register the type and extent of services that were claimed by this client group during the last six months prior to admission, and to calculate the costs that were caused. Further, we estimated the incremental costs for nursing home placement.

## Methods

### Setting

In Norway pension payments and specialist health care, including rehabilitation for substance abuse, are government-funded, while the responsibility for primary health care services and social services lies within the municipalities. Home care and support by social workers are issued by the municipalities and free of charge for the clients. The municipalities are required by law to offer accommodation adjusted to the clients’ housing abilities. This comprises housing programs for substance abusers, adjusted housing for physically or cognitive impaired persons, and, as the highest level of care, nursing homes. All of these measures require out-of pocket contributions. However, when a client has no financial means, social welfare will take over the costs until the client, eventually, might be able to pay back. As a measure for the client’s functioning in activities of daily living (ADL) the “IPLOS bistandsvariabler” questionnaire (individual-based care and support statistics, assistance variables) is used, containing 17 items that cover personal and instrumental ADL, hearing, and vision. Every item is scored from one to five, with higher scores indicating worse states, and the mean of all scores gives the total score [7].

### Study population

In May 2010 there were 17 residents at the addiction ward of Stokka Teaching Nursing Home. One criterion for admission to the addiction ward is a severe substance abuse, meaning that the alcohol or drug addiction is the main reason for the president’s health problems. At the time of study inclusion all residents used alcohol. Some used other drugs as well, but only to a small extent and not regularly. All of them had a history of longstanding alcohol or drug abuse for several decades. Most of them had started to have a problematic use of alcohol in their late teens or early twenties. Sixteen residents were invited to participate in the study, while one resident could not give informed consent, due to dementia. One resident chose not to participate.

### Municipal services

The following services are administered and financed by the municipality of Stavanger: Home care, cleaning help, day care centre, social workers, municipal housing, nursing homes and social welfare payments. Information about the use of municipal services was collected from the municipal registration system CosDoc [8]. CosDoc works as both an administrative and a medical file, registering all municipal care services rendered to the individual client and containing his medical files for home care and nursing home care including the IPLOS scores. Information about visits at the community-based emergency department (ED) was collected from its registration system WinMed [9]. As we could not get information about the exact charge for each visit, we used an average charge of €36. Information about social welfare payments was provided by the department for health and social care. We registered payments as net payments by deducting pay backs by the recipients during the same time period. Police custody at the local police office is as well financed by the municipality. Information about police custody was provided by the Stavanger police office as registered in “PolitiOperativt system”. As we were not able to identify the costs for one day in custody, we used the costs for one day in prison as a substitute. Kirkens Bymission is a private organization that renders social services to drug abusers like the possibility to earn a little money by doing odd jobs or selling a magazine. These services are refunded by the municipality. Information of services given to the study participants were collected directly at the organization.

### State-funded services

State-funded services included in-hospital stays and visits to the outpatient clinics at the Stavanger University Hospital, rehabilitation clinics for substance abuse and pension payments. Data about in-hospital stays and visits to the outpatient clinic were drawn from the files of the Stavanger University Hospital from the registry Nirvaco Medical Systems (NIMES). As this is the only hospital within a radius of two hours driving, we did not collect data from other hospitals. Costs for in-hospital stay and treatment in the outpatient department were estimated according to the Diagnosis-related group (DRG) refunding system. As the DRG system does not apply for stays at the mental ward, we estimated these costs based on average cost per in-hospital day which constituted €1182 per day (SAMDATA 2010). Data about pension payments were given by the Norwegian labour and welfare administration. We registered net payments after the deduction of taxes.

### Out of pocket payments

Municipal housing, overnight stays in hospices, and visits to the general practitioner or community-based ED are paid by the client. Data was collected from the municipal registration system CosDoc. In case the costs were covered by social welfare payments, they are registered as services paid by the municipality.

### Unit costs

A list of unit costs is given in Table 1, including the source of price information. All costs are calculated at a 2010 price level and expressed in Euro (€); 1€ = 8.26 Norwegian kroner (04.0.2010) [10].

**Table 1:** Unit costs

**Service**	**Costs per unit in €**	**Source of cost information**
Home cleaning	45,- per hour	Stavanger accountancy center,
Hjemmesykepleie	70,- per hour	Stavanger accountancy center
Social worker	49,- per hour	Stavanger accountancy center
Day care centre	45,- per day	Stavanger accountancy center
Nursing home	214,- per day	Stavanger accountancy center
Community-based ED	36,- per visit	Stavanger ED
Hospital, outpatient clinic	145,- per visit	Norwegian labour and welfare administration
In-hospital stay, somatic ward	According to DRG system	Hospital accountancy center
In-hospital stay, mental ward	1182,- per day	SAMDATA 2010
Specialized housing program for substance abusers	726,- per month	Department for rehabiliation, Stavanger
Municipal housing	716,- per month	Stavanger accountancy center
Hospice	69,- per night	Stavanger accountancy center
Treatment centre for substance abusers	605,- per day	Rogaland A-center
General practitioner	16,- per visit	Norwegian labour and welfare administration
	8,- per telephone contact	
Police custody	242,- per night	Used costs for prison term as substitute

*ED* = Emergency department; *DRG* = Diagnosis related groups.

### Viewpoints of the analysis

We described costs from the following viewpoints: Costs arising for the municipality, costs arising for the state, costs borne by the client, costs to society and total costs. Social welfare payments are included into costs to the municipality. Pension payments are included into costs to the state. To calculate costs borne by the client we deducted payments for specialized housing and hospice that were covered by social welfare. Costs to society do not include social welfare payments or pension payments as they are considered income transfers. Total costs include costs to the municipality, to the state and to the client.

### Incremental costs for admission to the addiction ward

We calculated the incremental cost for the admission to the addiction ward based on the assumption that the residents used the same amount of visits to the community-based ED, specialist health care and police custody as prior to admission. However, our clinical experience shows that in most residents the use of these services is markedly reduced after admission to the ward. We therefore included a sensitivity analysis, based on the assumption that only 50% of these services were used as compared to prior to admission and that no services outside the addiction ward were used.

### Statistics

The software program SPSS 15.0 (SPSS Inc; Chicago, USA) was used for statistical analysis. The correlations between IPLOS score and total costs respectively costs to the municipality were evaluated by Spearman’s rho correlation coefficient.

The study was approved by the Regional Committee for Medical Research Ethics, University of Bergen, Norway. All participants gave written consent.

## Results

Fifteen residents agreed to participate in the study. Mean age in May 2010 was 62.3 years (49 to 76 years), fourteen males and one female. Date of admission to the addiction ward was between July 2006 and May 2010. Four residents were admitted from their own homes, four came from a specialized housing program, three were transferred from other institutions and four had lived in hospices during the last weeks before admission. None of them was in an employment relationship, but only one had no income at all, while the others either received retirement or disability pension.

### Resource use prior to admission to nursing home

Services rendered, number of recipients and mean costs are shown in Table 2. During the last six months prior to admission the residents caused mean total costs of € 32 474 (€6080 to € 80 937) and median costs of €28 774 (interquartile range €21 555–€39 143). Of these costs 46.0% were borne by the municipality, 49.1% were state-funded and 5.0% were out-of-pocket payments.

**Table 2:** Services rendered, number of recipients and mean costs

	**Number of recipients of service**	**Mean use per recipient during study period**	**Median costs per recipient of service during study period in € (IQR)**	**Mean costs for all participants (n = 15) during study period in € (SD)**
**Servives paid by the municipality**				
Home cleaning	4	20 hours	869 (77–1738)	237 (525)
Home nursing	9	71.6 hours	4075 (419–9954)	3082 (4281)
Day care centre	1	26 days	1156	77 (299)
Nursing home	4	33 days	6750 (4500–10286)	1886 (3525)
Social worker	9	204.7 hours	12 097 (2688–12 097)	5827 (6092)
Police custody	7	6 nights	1453 (242–1937)	678 (1087)
Kirkens bymission	1		151	10 (39)
Social welfare	9		1639 (339–6466)	3266 (6309)
**Costs for municipal services**	**15**		**13 162 (5896–20 112)**	**15 064 (9525)**
**Municipal services paid by the client**				
Municipal housing	2	6 months	4298	573 (1512)
Specialized housing	6	5.14 months	1090 (0–2719)	812 (1554)
Hospice	3	16.3 days	0	74 (285)
General practitioner	10	8.5 visits	90 (41–100)	65 (79)
Community-based ED	10	5.1 visits	145 (73–218)	121 (169)
**Out-of-pocket payments**	**15**		**178 (82–2928)**	**1646 (21079)**
**State-funded services**				
In-hospital stay	6	23.1 days	7151 (1294–17 840)	6998 (14 171)
Outpatient clinic	3	18 visits	3588	521 (1862)
Treatment for substance abuse	1	15 days	9080	605 (2344)
Disability pension	9	6 months	8417 (6589–10 631)	5038 (4796)
Retirement pension	5	6 months	8333 (8250–8691)	2907 (4275)
**Costs for state-funded services**	**15**		**10 640 (8326–15 568)**	**16 069 (16 751)**
Pay back of debts	8		−251 (302–716)	−304 (–458)
**Total costs per six months**	**15**		**28 774 (21 555–39 143)**	**32 474 (18 284)**
**Minimum**				**6081**
**Maximum**				**80 937**

*SD* = standard deviation; *IQR* = Interquartile range where applicable.

One patient was admitted to a rehabilitation clinic for substance withdrawal. However, this measurement was taken due to the patient’s low housing abilities, and he was transferred to the addiction ward as soon as possible.

The mean functional level prior to admission as evaluated by the IPLOS- questionnaire was 3.01 (2.05 to 4.29). There was no significant correlation between these scores and total costs (rho = 0.17; p = 0.55) or between the IPLOS scores and costs to the municipality (rho = 0.22; p = 0.43).

### Incremental costs for placement at the addiction ward

Costs per bed at the addiction ward were € 38 196 for six months, 93% resident aimed costs and 7% maintenance costs. Residents have to contribute with out-of-pocket payments adjusted to their income. Assuming that all residents received minimum pension, 85% of this income (€ 6753) were transfer payments to the municipality while 15% (€ 1192) were incremental costs for pension payments. Assuming that residents at the addiction ward still used the same amount of visits to the community-based ED, specialist health care and police custody as prior to their admission, this would add up to additional costs of €8923 per six months. Total costs for residents of the addiction ward would thus be €47 120 per half year and the incremental costs €14 646. Assuming that the residents used only 50% of the services as compared to prior to admission, these costs would decrease to €42 658 and incremental costs of €10 184. Assuming no services outside the addiction ward were used total costs were €38 196 and incremental costs €5722 per six months. Table 3 shows absolute and incremental costs from the societal, national and municipal point of view, including the sensitivity analysis.

**Table 3:** Incremental costs and sensitivity analysis for admission to the addiction ward

**Viewpoint of ananlysis**	**Included costs**	**Costs prior to admission per 6 months in €**	**Estimated costs after admission per 6 months in €**	**Incremental costs per 6 months in €**
Assumed that the residents used the same resources than prior to admission*				
**Total costs**	Services paid by the municipality, the state and the client	32 474	47 120	+ 14 646
**National welfare system**	Specialist health care and pension payments	16 069	16 069	0
**Municipal welfare system**	Primary health care, social services and social welfare payment	15 064	38 991	+ 23 927
**Costs to society**	Primary and secondary health care and social services	22 212	39 097	+ 16 885
Assumed that the residents used 50% of the resources as prior to admission*				
**Total costs**	Services paid by the municipality, the state and the client	32 474	42 658	+ 10 184
**National welfare system**	Specialist health care and pension payments	16 069	12 310	–3759
**Municipal welfare system**	Primary health care, social services and social welfare payment	15 064	38 596	+ 23 531
**Costs to society**	Primary and secondary health care and social services	22 212	34 695	+ 12 483
Assumed that the residents used no services outside the addiction ward				
**Total costs**	Services paid by the municipality, the state and the client	32 474	38 196	+ 5722
**National welfare system**	Specialist health care and pension payments	16 069	7 945	–8124
**Municipal welfare system**	Primary health care, social services and social welfare payment	15 064	38 196	+23 132
**Costs to society**	Primary and secondary health care and social services	22 212	30 233	+ 8021

* Specialist health care, community-based ED and police custody.

## Discussion

In 15 persons with longstanding substance abuse the use of health care and social resources was registered during the six months prior to their admission to a specialized ward at a nursing home. Mean total costs during this period were € 32 474. Approximately half of these costs were borne by state-funded institutions, and half were borne by the municipality. We could show that these clients used a great variety of services aimed at subsistence, health care and support in independent living, while services aimed at drug withdrawal were not claimed. Our findings do not indicate any correlation between the amount of services rendered and the clients’ level of functioning. The incremental costs for nursing home admission were borne by the municipalities.

The strength of this study is a detailed and robust evaluation of sources for welfare in this client group. However, as the patient cohort consisted only of fifteen patients of one single municipality the results might not be generalizable. In addition, staffing costs are relatively high in Norway, and the pricing for the various services might therefore vary substantially in other countries. We applied 2010 prices, even if some services and payments were already rendered some years earlier, and we might thus have overestimated costs slightly. Further, we gave an estimate on incremental costs due to nursing home admission that range from €5722 to €14 646. Unfortunately, we have no detailed recording of the actual use of resources after admission that would allow for precise calculations. However, our clinical experience shows that in most residents the use of in- and outpatient hospital services is markedly reduced after admission to the nursing home as they are regularly seen by a permanently assigned doctor at the ward.

Costs caused by the residents prior to admission to the addiction ward showed a big variance with a maximum of €80 937 and a minimum of €6080. However, these costs might not reflect the actual health state and need for care of the clients, as there was no correlation between costs and functional level as measured by the IPLOS score. A study about the validity of the IPLOS score in evaluating the need for care in persons with dementia could show that this questionnaire was weak in assessing fluctuating presentations like neuropsychiatric symptoms [11]. This might apply for substance abusers, as well. In addition, a study on the general population 50 years and older in Western Europe showed that the use of health care services was positively related to the household income [12], and an US study found that patients with severe mental illness tended to seek care outside their primary care relationship [13]. This indicates that marginal client groups might marginalize themselves even further, which is as well reflected in our clinical experience. Clients who are already well-integrated in the welfare system have better access to further services and hence cause higher costs, while clients that have caused low costs prior to admission might simply not have sought for help due to low social skills or a tendency to avoid social contact. Thus, our study presents the actual costs caused by this client group, however, costs might be higher if all clients received services according to their disease severity.

There are few Nordic studies about the resource use and costs of persons with substance abuse, and most evaluations are reports with a nation-wide perspective [14-16]. To the best of our knowledge there are no previous studies with a similar cohort of persons with longstanding abuse. However, a US study about homeless people found mean annual costs for health care and social services of $7455 (about €5700), and the quintile with the highest resource use caused mean costs of $11 100 per person and year (about €8500), which are significantly lower numbers than our findings. Another Norwegian study about the use of municipal costs for health care services in persons with dementia found annual costs of about €28 000, including nursing home stay [17]. This suggests that both patient groups - persons with dementia and persons with longstanding substance abuse – can be considered high consumers of health care services, and the findings indicate that the costs caused to the health care system are in the same range.

We estimated the incremental costs for nursing home placement in order to set costs for health care and social services in perspective. However, one has to keep in mind that the placing in the addiction ward is not an economic decision. Main reason for admission is the loss of the ability to meet basic personal needs like nutrition and personal hygiene, and the aim of the treatment at the addiction ward is to stabilize physical and mental health while maintaining personal autonomy, and in consequence to improve the quality of life. In many ways these clients represent a fringe group as they are beyond the scope of rehabilitation and re-integration into a normal social life combined with a relatively short life expectancy. However, as substance abuse is an increasing problem, one might expect that this group will be growing over the next decades and require greater attention.

## Conclusion

We could show that persons with long standing substance abuse represent a group with a significant use of a great variety of health care and social services and hence cause high costs. However, our findings do not indicate any correlation between the amount of services rendered and the level of functioning. Further research should focus on the identification of the clients’ need for support in order to facilitate targeted interventions that might prevent further deterioration and, finally, the need for permanent care.

## Abbreviations

ADL: Daily life activities; IPLOS: Individual-based care and support statistics; ED: Emergency department; DRG: Diagnosis related groups.

## Competing interest

The authors declare that they have no competing interests.

## Authors’ contributions

CV participated in planning and designing the study, collected and analysed the data, and drafted the manuscript. IT participated in planning and designing the study and helped drafting the manuscript. RS participated in planning and designing the study and collecting the data. SN participated in planning and designing the study and helped drafting the manuscript. All authors read and approved the final manuscript.

## Authors’ information

RS is the head nurse of the addiction ward at Stokka Teaching Nursing Home, while CV is the physician for the residents of the ward.

## Pre-publication history

The pre-publication history for this paper can be accessed here:

http://www.biomedcentral.com/1472-6963/13/185/prepub
